# Advances in the Diagnosis and Monitoring of Hepatitis C Virus Infection

**DOI:** 10.4021/gr576e

**Published:** 2013-10-31

**Authors:** Marco Ciotti, Cartesio D’Agostini, Aldo Marrone

**Affiliations:** a Laboratory of Molecular Virology, Polyclinic Tor Vergata Foundation, Viale Oxford 81-00133, Rome, Italy; b Department of Experimental Medicine and Surgery, University of Rome “Tor Vergata”, Via Montpellier 1, 00133, Rome, Italy; c Laboratory of Clinical Microbiology and Virology, Polyclinic “Tor Vergata” Foundation, Viale Oxford 81, 00133, Rome, Italy; d Internal Medicine and Hepatology, School of Medicine of Naples, Second University of Naples, Via Pansini 5, Edificio 10, 80131, Napoli, Italy

**Keywords:** HCV genotypes, Quantitative HCV real-time, HCV serology, Antiviral therapy

## Abstract

Hepatitis C virus (HCV) infection represents a major health problem worldwide. Approximately 350,000 people die every year from hepatitis C related diseases. Antiviral therapy is given to prevent such complications. Advances in serological and molecular assays greatly improved the diagnosis of hepatitis C virus infection and the management of chronically infected patients. Sensitive real-time PCR methods are currently used to monitor the response to antiviral therapy, to guide treatment decisions, and to assess the sustained virological response 24 weeks after the end of therapy. HCV genotyping is part of the pretreatment evaluation. Determination of HCV genotype is important both for tailoring antiviral treatment and for determining treatment duration. It predicts also response to therapy. With the recent introduction of the serine protease inhibitors telaprevir and boceprevir, approved for the treatment of genotype 1 chronic hepatitis C in combination with INF-a and ribavirin, subtyping has become clinically relevant. Indeed, subtypes 1a and 1b may respond differently to current telaprevir-based or boceprevir-based triple therapy. This review summarizes the most recent advances in the diagnosis and monitoring of HCV chronic infection.

## Introduction

Hepatitis C virus (HCV) is a single stranded, positive sense, RNA virus of about 9.6 Kb in length. The genome consists of a conserved untraslated region (UTR) at both 5’ and 3’ termini which flanks a single open reading frame (ORF) encoding a polyprotein which is processed by cellular (signal peptidase and signal peptide peptidase) and viral proteases (NS2/3 and NS3/4A) to give rise to the single viral proteins: core, E1, E2, p7, NS2, NS3, NSA4A, NS4B, NS5A, NS5B [1]. The core, E1 and E2 proteins constitute the structural proteins and form the viral particle. The remaining proteins are non-structural and are involved in viral replication.

The virus belongs to the *Flaviviridae* family, genus *Hepacivirus*, and it is an important cause of chronic viral hepatitis. It is estimated that about 170 million people are chronically infected by HCV worldwide and 350,000 people die every year from hepatitis C-related liver diseases [2]. Antiviral therapy is given to avoid the progressive deterioration of liver function and the development of hepatocarcinoma.

HCV is characterized by high replication rate. It is estimated that about 10^12^ virions per day are produced in a given individual. This high replicative activity and the lack of proofreading activity of the RNA-dependent-RNA polymerase are responsible for the high genetic variability of the virus. These features are similar to those of HIV infection and provide the rationale for the development of new combination therapies.

Phylogenetic analysis of the core/E1, NS5B and complete genome sequences classified the HCV variants into six genetic groups called genotypes that differ from each other by 31-33% at nucleotide level. Each genetic group contains several subtypes which differ in their nucleotide sequence by 20-25%. The term quasispecies refer instead to the genetic heterogeneity of the population of HCV genomes that coexist within an infected individual [3, 4].

Determination of HCV genotypes is important to establish the duration of the antiviral therapy and for predicting the response to therapy. The actual standard of care therapy based on the administration of PEG-interferon and ribavirin requires 24 weeks of treatment for genotypes 2, 3, 5, and 6, and 48 weeks of treatment for genotypes 1 and 4. The response rate varies according to the HCV genotype. A sustained virological response (SRV) is achieved in 40-50% of the patients carrying HCV genotypes 1 and 4, and in about 80% of the patients infected with the other genotypes [5]. Nevertheless, it is unclear whether there is a correlation between HCV genotypes and disease severity or clinical outcome.

With the recent introduction of NS3/4 serine protease inhibitors, approved for treatment of patients infected by HCV genotype 1, the correct identification of HCV-1a and 1b subtypes has become clinically relevant [6-8] as well as the use of very sensitive assays for the detection of HCV RNA.

A quantitative real-time PCR with a lower limit of detection < 15 IU/mL and linear quantification down to this threshold is currently required for monitoring response to antiviral therapy and guide treatment decisions.

This review will focus and summarize the most recent advances in the diagnosis and monitoring of HCV infection.

### HCV life cycle

The HCV life cycle is very complex and not completely understood yet. Several molecules are implicated in the entry of HCV into the hepatocyte. Glucosaminoglycans and low-density lipoprotein receptors on the hepatocyte surface are believed to act as initial attachment factors, while scavenger receptor type B1 (SR-B1), CD81, and tight-junction proteins CLDN-1 (claudin-1) and OCLN (occludin) are involved in the uptake [9]. Once inside the cell, the virus is uncoated and the RNA genome released in the cytoplasm. The positive strand RNA is translated into a single large polyprotein which is cleaved by host and viral enzymes to generate 10 individual proteins; then it is copied into an intermediate negative strand RNA which is used to generate additional positive strand RNAs for subsequent rounds of translation and packaging into virus particles. The replication machinery includes the non structural proteins NS3, NS4A, NS4B, NS5A, and NS5B, and it is localized to the membranous web located in close contact with the perinuclear membranes. After encapsidation of the positive strand RNA genome, the viral particles are released from the infected cells. HCV uses the lipoprotein production pathway to assemble the viral particles and release them out of the infected cells [10-12].

*In vitro* systems such as HCV replicons are greatly contributing to the understanding of virus life cycle providing also the opportunity to test new candidate drugs in cell systems [13, 14].

## Diagnostic Methods

### Serological assays

#### HCV antibody assay (EIAs)

The serological diagnosis of HCV infection is based on the detection of specific anti-HCV antibodies. Starting from 1989, when the hepatitis C virus was first cloned [15], several immunoassays to detect HCV antibodies in serum or plasma specimens have been developed. The first generation assay was based on a yeast-expressed recombinant protein containing the epitope C100-3 from the NS4 region of the HCV genome [16]. With the first generation assay, the level of false positive results was quite high. Therefore, it was necessary to improve the serological screening with the introduction of more advanced assays. The second generation assay used a multiantigen format which included antigens from the core, NS3 and NS4 regions. These modifications markedly improved sensitivity and specificity of the assay [17]. However, differences in serologic reactivity to HCV antigens among different HCV genotypes were reported [18]. The third generation assay included an additional antigen from the NS5 region [19]. The third generation assays have a higher sensitivity and specificity than second generation assays and are much less strongly influenced by the HCV genotype [20-22]. In addition, the third generation assay reduced the window period by an average of 5 weeks compared to the first generation assay and detected anti-HCV antibodies as early as 10 weeks after exposure. The diagnostic specificity of current HCV third generation assays is > 99% [23]. However, it is still possible to observe false positive results (for example. patients with autoimmune diseases, mononucleosis, pregnancy), while false negative results may occur in subjects with severe immunosuppression such as HIV infection, hypo- or agammaglobulinemia, solid organ transplant recipients, or in patients on hemodialysis [24-27].

#### Immunoblot assay

The recombinant immunoblot antibody assay (RIBA) is a test which detects antibodies to HCV in human serum or plasma. It is intended for use as a supplementary confirmatory test on human serum or plasma specimens found to be reactive to HCV using an anti-HCV screening procedure. Detection of anti-HCV by RIBA is based on immobilization of HCV recombinant antigens and synthetic peptides from the core, the E2 hypervariable region (HVR), the NS3 helicase region, the NS4A, NS4B and NS5A regions (INNO-LIA™ HCV Score, Innogenetics, Gent, Belgium). A sample is considered positive for HCV antibodies if at least two protein lines are reactive; if only one protein line reacts, then the result is considered indeterminate. In HCV infected individuals, the assays are generally indeterminate during the first weeks of infection and become fully positive 1 - 6 months later [28]. Persistent RIBA-indeterminate reactions are usually indicative of recovery from a remote HCV infection [29].

A true positive RIBA test indicates only the presence of anti-HCV antibodies and may reflect past infection with spontaneous clearance. Therefore, confirmation of active infection still requires HCV RNA testing that overshadowed the role of RIBA testing in HCV diagnosis [30]

#### HCV core antigen assay

As stated above, the virological diagnosis of HCV is based on the detection of anti-HCV antibodies. However, the antibody test does not discriminate between acute, past and persistent infections. Thus, amplification of HCV RNA by sensitive PCR is regarded as the method of choice to confirm an active infection in the immunocompetent individuals with positive anti-HCV test or in immunocompromised patients who could not mount an antibody response to HCV.

In recent years, automated quantitative HCV core antigen tests were developed and growing evidences show that they may be a useful alternative to HCV RNA testing [31-33]. They can detect HCV core antigen during the window period of acute infection although low titer RNA samples can be missed [34, 35].

A strong correlation between serum HCV core antigen level and viremia has been reported in patients with chronic C hepatitis [36]; and it has been suggested that quantification of total HCV core antigen may constitute a useful alternative to HCV-RNA measurement for predicting and monitoring response to PEG-IFN/Ribavirin treatment [37-42].

Worth of note, the amount of HCV core protein in serum might be a significant factor for determining histological outcome in patients with chronic hepatitis C [43].

One limitation of the HCV core antigen assay is that it has lower sensitivity than NAT. Thus, blood screening by HCV core antigen assay it is not recommended when the PCR methods are available. Instead, it might be used in low resource settings.

### Diagnosis of acute HCV infection

#### IgM anti-HCV and avidity

Acute HCV infection refers to the presence of clinical signs or symptoms of hepatitis for a period of 6 months or shorter after the presumed exposure to the virus. The majority of patients with acute hepatitis C are asymptomatic, but in about 50-90% of the cases infection becomes chronic.

Early diagnosis is important because an early treatment with PEG-IFN-α monotherapy can lead to a SVR in up to 90% of the cases [4, 44].

Diagnosis of acute HCV infection is frequently missed and the gold standard for diagnosis includes anti-HCV seroconversion or HCV RNA positivity or both. In recent years, efforts have been addressed towards the diagnosis of acute infection. IgM antibodies against HCV can be detected during acute and chronic phases. Therefore anti-HCV IgMs cannot be used as a diagnostic marker of acute HCV infection. As such, no anti-HCV IgM assays are licensed for clinical use.

Several studies instead have investigated whether HCV avidity assay can be used to improve acute hepatitis C diagnosis and distinguish between chronic and recent infection [45-50]. Antibody avidity is the binding capacity of maturing antibody with antigen, which increase over time. A dissociation agent can remove weakly bound antibody [51]. Antibodies generated early in infection have weak antigen-binding capacity compared to a matured antibody generated against the same antigen. It has been shown that testing for antibody avidity IgG and anti-HCV immunoglobulin M in a single serum sample allow diagnosis in up to 90% of cases of acute hepatitis C [52, 53]. Furthermore, these studies suggest that avidity test can be used both to enhance HCV surveillance and to identify individuals with acute infection who would benefit from early HCV treatment. However, these promising assays require further evaluation and validation in various clinical setting.

### Determination of hepatitis C genotype

As mentioned above, HCV isolates are classified into six genotypes which show a characteristic geographic distribution. Genotype 1, subtypes 1a and 1b as well as genotype 2, subtypes 2a, 2b, and 2c represent the most common variants in Western countries. Genotype 3 is widely distributed in South and East Asia with the subtype 3a common among intravenous drug users from Europe; genotype 4 in North Africa and Middle-East; genotype 5 in South Africa, and genotype 6 in Asia [54]. HCV genotyping is part of the pretreatment evaluation, and it is an important factor both for tailoring antiviral treatment and for determining treatment duration. For quite long time, genotyping methods targeted the 5’-untraslated region of HCV genome. Although this region is highly conserved among HCV variants, inside the region there are well characterized nucleotide polymorphisms that allow an accurate identification of the HCV genotypes in the majority of the cases. These polymorphisms can be detected by probe hybridization [55], restriction enzyme digestion [56] or direct sequencing [57].

Currently, the commercial assays available on the market for HCV genotyping include: 1) the INNO-LiPA HCV v.2.0 (Siemens Healthcare Diagnostics, Eragny, France); 2) The TRUGENE HCV Genotyping Assay (Siemens Healthcare Diagnostics, Eragny, France); 3) the Abbott Real-Time HCV Genotype II assay (Abbott Molecular, Des Plaines, IL, USA).

The INNO-LiPA is a reverse hybridization assay which targets the 5’UTR and the core region of the HCV genome. The inclusion of the core region was necessary because the sole interrogation of the 5’UTR did not guarantee an accurate discrimination between subtypes 1a and 1b and between genotypes 1 and 6 [58-60]. The Trugene kit uses the direct sequence analysis of the 5’UTR to genotype HCV [61], while the Abbott Real-Time HCV Genotype II assay targets the 5’UTR and the NS5B gene for efficient discrimination of subtypes 1a and 1b [62]. With the introduction of the new anti-HCV drugs telaprevir and boceprevir, two serine protease inhibitors approved for treatment of patients infected by HCV genotype 1, the efficient discrimination between subtypes 1a and 1b has become mandatory [6].

### Detection and monitoring of HCV RNA level

About 85% of the people with acute infection will become chronic carriers [63]. Determination of antibody response and detection of HCV RNA are both essential for diagnosis of chronic HCV infection. Monitoring HCV RNA level is crucial for the management of patients on antiviral treatment. Over the years, several molecular methods have been developed for measuring the HCV RNA level in serum such as the branched DNA assay based on the signal amplification; and the end-point quantitative reverse-transcription PCR (RT-PCR) assays based on the amplification of the 5’ UTR sequence [64]. At present, real-time RT-PCR is the method of choice for measuring HCV RNA level in serum samples and for the management of HCV chronic patients. Compared to previous assays, real-time RT-PCR offers a series of advantages such as 1) increased analytical sensitivity (10 - 15 IU/mL), 2) faster results, 3) reduced risk of contamination, and 4) wider dynamic range (up to 7 - 8 log IU/mL). Currently, four real-time PCR assays are available on the market for the routine diagnostic determination of HCV viral load: the Cobas Ampliprep/Cobas TaqMan HCV v. 2 assay [65] (CAP/CTM HCV v.2.0, Roche Molecular System, Pleasanton, CA), the Abbott RealTime HCV assay [65] (Abbott Diagnostics, Chicago, IL), the VERSANT HCV RNA 1.0 assay (kPCR) (Siemens Healthcare Diagnostics Inc., Tarrytown, NY, USA), and the Artus HCV QS-RGQ kit [66] (Qiagen, Hilden, Germany) (Table 1). The performance characteristics of the CAP/CTM HCV v. 2.0 assay are significantly improved compared to the CAP/CTM HCV v.1.0 which underestimated about 15% of HCV genotype 2 samples and 30% of genotype 4 samples and overestimated samples with genotypes 1, 3, 5, and 6 [67, 68]. In a recent study the CAP/CTM HCV v. 2.0 was compared to the Abbott RealTime HCV assay and the performance characteristics of the Roche assay were comparable to those of the Abbott assay [65].

**Table 1:** Technical Features of Diagnostic Real-time HCV Assays Available Currently on the Market

Technical features	CAP/CTM HCV v.2 (Roche diagnostics)	Real-time HCV (Abbott molecular)	*Artus* HCV QS-RGQ v.1 (Qiagen)	VERSANT HCV RNA 1.0 assay (kPCR) (Siemens Health Care)
LOD (IU/mL)*	15	12	20	15
Specificity	100%	100%	100%	100%
Linear range (IU/mL)	15 - 1 × 10^8^	12 - 1 × 10^8^	25 - 1.77 × 10^7^	15 - 1 × 10^8^
Genotypes	1 - 6	1 - 6	1 - 6	1 - 6

* Probit analysis: 2nd WHO International Standard (96/798) for Roche; 1th WHO Standard 96/790 for Abbott; Acrometrix (2nd WHO std) for Qiagen; WHO 3rd International Standard (60/100) for Siemens.

While quantitative detection is important for determining the basal viral load and for monitoring treatment response at 4, 12 and 24 weeks, sensitive qualitative detection is essential both for confirming active infection and assessing viral clearance in response to treatment [69].

To this end, transcription-mediated amplification method (TMA, Siemens Healthcare Diagnostics Inc., Tarrytown, NY, USA) and real-time RT-PCR with a limit of detection of at least 15 IU/mL perform both well, show comparable sensitivity and equivalent genotype reactivity [65, 69-71]. However, real-time PCR is progressively replacing the other methods because of easy to use, complete automation and high sensitivity.

According to the EASL guidelines, a sustained virological response is defined as an undetectable HCV RNA level (< 50 IU/mL) 24 weeks after treatment withdrawal [5]. Therefore, patients on antiviral treatment with PEG-IFN plus ribavirin should be monitored with a PCR method which has a limit of quantification of 50 IU/mL or less [5]. With the approval by FDA (Food and Drug Administration, USA) and EMA (European Medicine Agency, EU) of the directly acting anti-viral agents (DAAs) telaprevir and boceprevir, two NS3/4A protease inhibitors, the limits of quantification (LOQ) and detection (LOD) changed as well as the time points for monitoring treatment response. A LOQ of 25 IU/mL and a LOD of 10 IU/mL are required for the correct management of patients treated with NS3/4A protease inhibitors [7, 8].

### Endpoints of HCV therapy and virological response guided therapy

The ultimate goal of HCV therapy is the eradication of the infection in order to prevent the complications of HCV related liver diseases including necroinflammation, fibrosis, cirrhosis, HCC, and death. So, the primary endpoint of HCV therapy is the sustained virological response (SVR), while intermediate endpoints are used during the standard of care treatment (SoC) (PEG-IFN + ribavirin) to assess the likelihood of SVR and determine treatment duration.

Sensitive real-time PCR methods with a lower limit of quantification of 50 IU/mL are suggested to monitor viral RNA kinetics in response to antiviral therapy. The same method must be used during monitoring to ensure comparability.

The intermediate endpoints require HCV RNA measurement at 4, 12 and 24 weeks to identify the rapid virological response (RVR), early virological response (EVR) and delayed virological response (DVR), respectively. The likelihood of SVR is directly proportional to the time of HCV RNA disappearance. Patients who achieve an undetectable HCV RNA at 4 weeks of treatment have a SVR rate of approximately 85-90% and these patients could benefit from a shorter period of treatment of 24 weeks [72-75]. So, response guided therapy (RGT) to SOC and stopping rules are established on the basis of HCV RNA drop or detection at different time points, respectively. Null response (NR) is characterized by less than 2 log_10_ IU/mL decrease in HCV RNA level from baseline at 12 weeks of therapy, whereas partial response (PR) by more than 2 log_10_ IU/ml decrease in HCV RNA level from baseline at 12 weeks of therapy but with detectable HCV RNA at weeks 12 and 24 [5, 76]. However, the HCV treatment terminology has been updated after the introduction of the two new approved direct-acting antiviral (DAA) medications given in combination with PEG-IFN/RBV. The lead-in phase (4 weeks of PEG-IFN/RBV before boceprevir adding) and the extended rapid virological response (eRVR) (unquantifiable HCV RNA at week 4 and through week 12 of therapy) have been included for a better evaluation of the virological response guided therapy in patients receiving a triple regimen treatment which includes boceprevir or telaprevir [76-79].

In patients infected with HCV genotype 1, an SVR is obtained in about 40-54% of patients treated with with PEG-IFN/RBV at approved doses for 48 weeks, and in 65-82% of patients infected with HCV genotypes 2 or 3 treated with PEG-IFN/RBV at approved doses for 24 weeks [5].

In patients receiving SOC, treatment for all HCV genotypes should be stopped at week 12 if the HCV RNA decrease is less than 2 log_10_ IU/ mL and at week 24 if HCV RNA is still detectable (≥ 50 IU/mL) [5].

With the introduction of the protease inhibitors, boceprevir and telaprevir, SVR rates increased up to 79% when used as triple therapy regimen in treatment-naive and in previous relapse patients with genotype 1 [5, 76, 80, 81].

In patients receiving triple therapy, the following stopping rules have been established on the basis of virological response. In naive patients: 1) treatment with boceprevir, PEG-IFN/RBV should be stopped if the HCV RNA level is > 100 IU/mL at treatment week 12 or detectable at treatment week 24; 2) treatment with telaprevir, PEG-IFN/RBV should be stopped if the HCV RNA level is > 1,000 IU/mL at treatment weeks 4 or 12 and/or detectable at treatment week 24.

In experienced patients: 1) patients re-treated with boceprevir plus PEG-IFN/RBV who continue to have detectable HCV RNA > 100 IU at week 12 should be withdrawn from all therapy because of the high likelihood of developing antiviral resistance; 2) patients re-treated with telaprevir plus PEG-IFN/RBV who continue to have detectable HCV RNA > 1,000 IU at weeks 4 or 12 should be withdrawn from all therapy because of the high likelihood of developing antiviral resistance [76].

### *IL28B* gene polymorphisms

It is known that viral eradication in treated patients is influenced by both viral and host factors. For instance, high viral load at baseline, viral genotype 1, high body mass index, insulin resistance and severe fibrosis affect negatively the response to antiviral therapy [82]. Among the host factors, ethnicity plays a role in predicting response to antiviral therapy. African-Americans present a lower response rate to PEG-IFNa than Caucasians [83]. Nucleotide polymorphisms (SNPs) upstream of the *IL28B* gene can predict hepatitis C virus persistence and response to antiviral therapy. For instance, it was found that polymorphism rs12979860 located on chromosome 19 is strongly associated with SVR in patients treated with PEG-IFN and ribavirin [84]. This polymorphism resides 3 kb upstream of the *IL28B* gene which encodes for the IFN-l3.

In all ethnic groups examined (European-Americans, African-Americans and Hispanics) the CC allele was associated with a higher rate of SVR than the CT and TT alleles [84]. The frequency of the CC allele varies among different ethnic groups. It is the highest among East Asians, and lowest among African-Americans. The rate of SVR is in good concordance with the presence of the CC allele. Indeed, a higher rate of SVR has been observed in African-Americans with the CC allele compared with the individuals of European ancestry with the TT allele [84].

With the addition of the protease inhibitors to INF-a and ribavirin, SVR rates improved for all IL28B genotypes. This seems to attenuate the importance of IL28B genotype in the presence of triple drugs regimens since unfavorable IL28B genotypes benefit from the addition of the protease inhibitors. However, because hepatitis C treatment is moving towards interferon-free regimens, the role of IL28B in these future regimens needs to be further investigated [80, 85].

### HCV drug resistance

In the last few years several novel molecules targeting specific viral proteins involved in the HCV life cycle have been developed. Among these, the NS3/4A protease inhibitors telaprevir and boceprevir have been approved for treating HCV chronic patients infected by genotype 1. These drugs when given in combination with PEG-INF/RBV improved significantly treatment outcome [7, 8, 86-90]. However, resistance develops quickly because of the high replication rate of the virus and the lack of proofreading activity of the NS5B RNA-dependent RNA polymerase that result in the generation of mutations that can affect the sensitivity of the virus to these compounds. Thus, the long term effectiveness of such drugs is challenged by the emergence of resistant variant strains [91, 92].

Differences in the sensitivity to boceprevir and telaprevir have been observed at genotype and subtype level [92, 93]. Looking at the subtype level, resistant variants and viral breakthrough have been observed consistently more frequently in patients infected with HCV subtype 1a than subtype 1b [86, 94]. This difference was shown to result from nucleotide differences at position 155 in HCV subtype 1a *versus* 1b. The mutation most frequently associated with resistance to telaprevir is R155K. This amino acid substitution requires 1 nucleotide change in HCV subtype 1a and 2 nucleotide changes in subtype 1b isolates. This difference between subtypes 1a and 1b in the selection of the resistant variant R155K was observed also for boceprevir [95].

Mutations at six amino acid positions (V36A/M, T54S/A, V55A, R155K/T/Q, A156T/V, V170A/T) are generally associated with resistance to telaprevir and boceprevir [92] while macrocyclic inhibitors more commonly select for D168A/V/T/H and R155K/T/Q variants [96]

The S282T mutation has been detected in the NS5B gene of one patient with HCV genotype 2b infection treated with sofosbuvir-monotherapy. No mutation at 282 positions was found in the other three patients on the same treatment regimen as well as in previously untreated patients infected with HCV genotype 1 or patients infected with genotype 1 who did not respond to prior treatment [97]. This mutation warrants further investigations in future clinical studies.

Considering the similarity between HIV and HCV in terms of high genetic variability and high replication rate, resistance profiling will remain an issue for the next generation protease inhibitors and probably also for the other classes of drugs waiting for approval.

The use of drugs with different mechanisms of action will be probably the best strategy to prevent resistance and increase the chance to eradicate HCV infection.

## Conclusions

Hepatitis C chronic infection represents a major health problem worldwide and has a great socioeconomic impact. Advances in serological and molecular diagnosis of HCV infection improved greatly the management of patients infected by this virus. Nowdays, sensitive HCV real-time PCRs are available and guide the response to treatment. New therapeutic algorithms have been derived based on these new technologies, and they are used to tailor treatment regimens and to stop therapy when the likelihood of a sustained virological response is null.

Accurate HCV genotyping/subtyping is crucial for the correct management of patients with HCV chronic infection. Different genotype/sybtypes may show different sensitivity to the drugs used with possible consequences on treatment outcome.

## References

[1] Major ME, Rehermann B, Feinstone SM, Knipe DM, Peter M, Howley PM (2001). Hepatitis C viruses.

[2] http://www.who.int/mediacentre/factsheets/fs164/en/

[3] Marrone A, Sallie R (1996). Genetic heterogeneity of hepatitis C virus. The clinical significance of genotypes and quasispecies behavior. Clin Lab Med.

[4] Simmonds P, Bukh J, Combet C, Deleage G, Enomoto N, Feinstone S, Halfon P (2005). Consensus proposals for a unified system of nomenclature of hepatitis C virus genotypes. Hepatology.

[5] (2011). EASL Clinical Practice Guidelines: management of hepatitis C virus infection. J Hepatol.

[6] Chevaliez S, Bouvier-Alias M, Brillet R, Pawlotsky JM (2009). Hepatitis C virus (HCV) genotype 1 subtype identification in new HCV drug development and future clinical practice. PLoS One.

[7] Poordad F, McCone J, Bacon BR, Bruno S, Manns MP, Sulkowski MS, Jacobson IM (2011). Boceprevir for untreated chronic HCV genotype 1 infection. N Engl J Med.

[8] Jacobson IM, McHutchison JG, Dusheiko G, Di Bisceglie AM, Reddy KR, Bzowej NH, Marcellin P (2011). Telaprevir for previously untreated chronic hepatitis C virus infection. N Engl J Med.

[9] Rice CM (2011). New insights into HCV replication: potential antiviral targets. Top Antivir Med.

[10] Herker E, Ott M (2011). Unique ties between hepatitis C virus replication and intracellular lipids. Trends Endocrinol Metab.

[11] Wakita T, Pietschmann T, Kato T, Date T, Miyamoto M, Zhao Z, Murthy K (2005). Production of infectious hepatitis C virus in tissue culture from a cloned viral genome. Nat Med.

[12] Da Costa D, Turek M, Felmlee DJ, Girardi E, Pfeffer S, Long G, Bartenschlager R (2012). Reconstitution of the entire hepatitis C virus life cycle in nonhepatic cells. J Virol.

[13] Zhong J, Gastaminza P, Cheng G, Kapadia S, Kato T, Burton DR, Wieland SF (2005). Robust hepatitis C virus infection in vitro. Proc Natl Acad Sci U S A.

[14] Lindenbach BD, Evans MJ, Syder AJ, Wolk B, Tellinghuisen TL, Liu CC, Maruyama T (2005). Complete replication of hepatitis C virus in cell culture. Science.

[15] Choo QL, Kuo G, Weiner AJ, Overby LR, Bradley DW, Houghton M (1989). Isolation of a cDNA clone derived from a blood-borne non-A, non-B viral hepatitis genome. Science.

[16] Kuo G, Choo QL, Alter HJ, Gitnick GL, Redeker AG, Purcell RH, Miyamura T (1989). An assay for circulating antibodies to a major etiologic virus of human non-A, non-B hepatitis. Science.

[17] Alter HJ (1992). New kit on the block: evaluation of second-generation assays for detection of antibody to the hepatitis C virus. Hepatology.

[18] Zein NN, Rakela J, Persing DH (1995). Genotype-dependent serologic reactivities in patients infected with hepatitis C virus in the United States. Mayo Clin Proc.

[19] Barrera JM, Francis B, Ercilla G, Nelles M, Achord D, Darner J, Lee SR (1995). Improved detection of anti-HCV in post-transfusion hepatitis by a third-generation ELISA. Vox Sang.

[20] Kao JH, Lai MY, Hwang YT, Yang PM, Chen PJ, Sheu JC, Wang TH (1996). Chronic hepatitis C without anti-hepatitis C antibodies by second-generation assay. A clinicopathologic study and demonstration of the usefulness of a third-generation assay. Dig Dis Sci.

[21] Maggi F, Vatteroni ML, Pistello M, Avio CM, Cecconi N, Panicucci F, Bendinelli M (1995). Serological reactivity and viral genotypes in hepatitis C virus infection. J Clin Microbiol.

[22] Makris K, Kouvelis V, Drakopoulos I, Oikonomou E, Maniatis A (1995). Frequency and characteristics of post-transfusion hepatitis in Greece with emphasis on hepatitis C: comparing second- and third-generation assays. Transfus Med.

[23] Colin C, Lanoir D, Touzet S, Meyaud-Kraemer L, Bailly F, Trepo C (2001). Sensitivity and specificity of third-generation hepatitis C virus antibody detection assays: an analysis of the literature. J Viral Hepat.

[24] Zhu WF, Lei SY, Li LJ (2011). Hepatitis C virus infection and biological false-positive syphilis test: a single-center experience. Hepatobiliary Pancreat Dis Int.

[25] Senevirathna D, Amuduwage S, Weerasingam S, Jayasinghe S, Fernandopulle N (2011). Hepatitis C virus in healthy blood donors in Sri Lanka. Asian J Transfus Sci.

[26] Thio CL, Nolt KR, Astemborski J, Vlahov D, Nelson KE, Thomas DL (2000). Screening for hepatitis C virus in human immunodeficiency virus-infected individuals. J Clin Microbiol.

[27] Kalantar-Zadeh K, Miller LG, Daar ES (2005). Diagnostic discordance for hepatitis C virus infection in hemodialysis patients. Am J Kidney Dis.

[28] Gerlach JT, Diepolder HM, Zachoval R, Gruener NH, Jung MC, Ulsenheimer A, Schraut WW (2003). Acute hepatitis C: high rate of both spontaneous and treatment-induced viral clearance. Gastroenterology.

[29] Makuria AT, Raghuraman S, Burbelo PD, Cantilena CC, Allison RD, Gibble J, Rehermann B (2012). The clinical relevance of persistent recombinant immunoblot assay-indeterminate reactions: insights into the natural history of hepatitis C virus infection and implications for donor counseling. Transfusion.

[30] Ghany MG, Strader DB, Thomas DL, Seeff LB (2009). Diagnosis, management, and treatment of hepatitis C: an update. Hepatology.

[31] Muerhoff AS, Jiang L, Shah DO, Gutierrez RA, Patel J, Garolis C, Kyrk CR (2002). Detection of HCV core antigen in human serum and plasma with an automated chemiluminescent immunoassay. Transfusion.

[32] Bouvier-Alias M, Patel K, Dahari H, Beaucourt S, Larderie P, Blatt L, Hezode C (2002). Clinical utility of total HCV core antigen quantification: a new indirect marker of HCV replication. Hepatology.

[33] Kesli R, Polat H, Terzi Y, Kurtoglu MG, Uyar Y (2011). Comparison of a newly developed automated and quantitative hepatitis C virus (HCV) core antigen test with the HCV RNA assay for clinical usefulness in confirming anti-HCV results. J Clin Microbiol.

[34] Nubling CM, Unger G, Chudy M, Raia S, Lower J (2002). Sensitivity of HCV core antigen and HCV RNA detection in the early infection phase. Transfusion.

[35] Tobler LH, Stramer SL, Lee SR, Baggett D, Wright D, Hirschkorn D, Walsh I (2005). Performance of ORTHO HCV core antigen and trak-C assays for detection of viraemia in pre-seroconversion plasma and whole blood donors. Vox Sang.

[36] Tanaka E, Ohue C, Aoyagi K, Yamaguchi K, Yagi S, Kiyosawa K, Alter HJ (2000). Evaluation of a new enzyme immunoassay for hepatitis C virus (HCV) core antigen with clinical sensitivity approximating that of genomic amplification of HCV RNA. Hepatology.

[37] Maynard M, Pradat P, Berthillon P, Picchio G, Voirin N, Martinot M, Marcellin P (2003). Clinical relevance of total HCV core antigen testing for hepatitis C monitoring and for predicting patients' response to therapy. J Viral Hepat.

[38] Vermehren J, Susser S, Berger A, Perner D, Peiffer KH, Allwinn R, Zeuzem S (2012). Clinical utility of the ARCHITECT HCV Ag assay for early treatment monitoring in patients with chronic hepatitis C genotype 1 infection. J Clin Virol.

[39] Descamps V, Op de Beeck A, Plassart C, Brochot E, Francois C, Helle F, Adler M (2012). Strong correlation between liver and serum levels of hepatitis C virus core antigen and RNA in chronically infected patients. J Clin Microbiol.

[40] Tedder RS, Tuke P, Wallis N, Wright M, Nicholson L, Grant PR (2013). Therapy-induced clearance of HCV core antigen from plasma predicts an end of treatment viral response. J Viral Hepat.

[41] Loggi E, Cursaro C, Scuteri A, Grandini E, Panno AM, Galli S, Furlini G (2013). Patterns of HCV-RNA and HCV core antigen in the early monitoring of standard treatment for chronic hepatitis C. J Clin Virol.

[42] Zanetti AR, Romano L, Brunetto M, Colombo M, Bellati G, Tackney C (2003). Total HCV core antigen assay: a new marker of hepatitis C viremia for monitoring the progress of therapy. J Med Virol.

[43] Iijima A, Tanaka E, Kobayashi M, Yagi S, Mizokami M, Kiyosawa K (2000). Relationship between histological prognosis of chronic hepatitis C and amount of hepatitis C virus core protein in serum. J Gastroenterol Hepatol.

[44] Santantonio T, Wiegand J, Gerlach JT (2008). Acute hepatitis C: current status and remaining challenges. J Hepatol.

[45] Shepherd SJ, Kean J, Hutchinson SJ, Cameron SO, Goldberg DJ, Carman WF, Gunson RN (2013). A hepatitis C avidity test for determining recent and past infections in both plasma and dried blood spots. J Clin Virol.

[46] Ward KN, Dhaliwal W, Ashworth KL, Clutterbuck EJ, Teo CG (1994). Measurement of antibody avidity for hepatitis C virus distinguishes primary antibody responses from passively acquired antibody. J Med Virol.

[47] Kanno A, Kazuyama Y (2002). Immunoglobulin G antibody avidity assay for serodiagnosis of hepatitis C virus infection. J Med Virol.

[48] Coppola N, Pisapia R, Marrocco C, Martini S, Vatiero LM, Messina V, Tonziello G (2007). Anti-HCV IgG avidity index in acute hepatitis C. J Clin Virol.

[49] Klimashevskaya S, Obriadina A, Ulanova T, Bochkova G, Burkov A, Araujo A, Stramer SL (2007). Distinguishing acute from chronic and resolved hepatitis C virus (HCV) infections by measurement of anti-HCV immunoglobulin G avidity index. J Clin Microbiol.

[50] Gaudy-Graffin C, Lesage G, Kousignian I, Laperche S, Girault A, Dubois F, Goudeau A (2010). Use of an anti-hepatitis C virus (HCV) IgG avidity assay to identify recent HCV infection. J Clin Microbiol.

[51] Dimitrov JD, Lacroix-Desmazes S, Kaveri SV (2011). Important parameters for evaluation of antibody avidity by immunosorbent assay. Anal Biochem.

[52] Coppola N, Pisapia R, Tonziello G, Masiello A, Martini S, Pisaturo M, Messina V (2009). Improvement in the aetiological diagnosis of acute hepatitis C: a diagnostic protocol based on the anti-HCV-IgM titre and IgG Avidity Index. J Clin Virol.

[53] Sagnelli E, Tonziello G, Pisaturo M, Sagnelli C, Coppola N (2012). Clinical applications of antibody avidity and immunoglobulin M testing in acute HCV infection. Antivir Ther.

[54] Kuiken C, Simmonds P (2009). Nomenclature and numbering of the hepatitis C virus. Methods Mol Biol.

[55] Stuyver L, Rossau R, Wyseur A, Duhamel M, Vanderborght B, Van Heuverswyn H, Maertens G (1993). Typing of hepatitis C virus isolates and characterization of new subtypes using a line probe assay. J Gen Virol.

[56] Nakao T, Enomoto N, Takada N, Takada A, Date T (1991). Typing of hepatitis C virus genomes by restriction fragment length polymorphism. J Gen Virol.

[57] Nolte FS, Green AM, Fiebelkorn KR, Caliendo AM, Sturchio C, Grunwald A, Healy M (2003). Clinical evaluation of two methods for genotyping hepatitis C virus based on analysis of the 5' noncoding region. J Clin Microbiol.

[58] Chen Z, Weck KE (2002). Hepatitis C virus genotyping: interrogation of the 5' untranslated region cannot accurately distinguish genotypes 1a and 1b. J Clin Microbiol.

[59] Bouchardeau F, Cantaloube JF, Chevaliez S, Portal C, Razer A, Lefrere JJ, Pawlotsky JM (2007). Improvement of hepatitis C virus (HCV) genotype determination with the new version of the INNO-LiPA HCV assay. J Clin Microbiol.

[60] Verbeeck J, Stanley MJ, Shieh J, Celis L, Huyck E, Wollants E, Morimoto J (2008). Evaluation of Versant hepatitis C virus genotype assay (LiPA) 2.0. J Clin Microbiol.

[61] Roque-Afonso AM, Ferey MP, Poveda JD, Marchadier E, Dussaix E (2002). Performance of TRUGENE hepatitis C virus 5' noncoding genotyping kit, a new CLIP sequencing-based assay for hepatitis C virus genotype determination. J Viral Hepat.

[62] Ciotti M, Marcuccilli F, Guenci T, Babakir-Mina M, Chiodo F, Favarato M, Perno CF (2010). A multicenter evaluation of the Abbott RealTime HCV Genotype II assay. J Virol Methods.

[63] Seeff LB (2002). Natural history of chronic hepatitis C. Hepatology.

[64] Pawlotsky JM (2002). Molecular diagnosis of viral hepatitis. Gastroenterology.

[65] Pas S, Molenkamp R, Schinkel J, Rebers S, Copra C, Seven-Deniz S, Thamke D (2013). Performance evaluation of the new Roche cobas AmpliPrep/cobas TaqMan HCV test, version 2.0, for detection and quantification of hepatitis C virus RNA. J Clin Microbiol.

[66] Paba P, Fabeni L, Perno CF, Ciotti M (2012). Performance evaluation of the Artus hepatitis C virus QS-RGQ assay. J Virol Methods.

[67] Chevaliez S, Bouvier-Alias M, Brillet R, Pawlotsky JM (2007). Overestimation and underestimation of hepatitis C virus RNA levels in a widely used real-time polymerase chain reaction-based method. Hepatology.

[68] Chevaliez S, Fix J, Soulier A, Pawlotsky J-M (2009). Underestimation of Hepatitis C Virus Genotype 4 RNA Levels by the Cobas AmpliPrep/Cobas TaqMan Assay. Hepatology.

[69] Hendricks DA, Friesenhahn M, Tanimoto L, Goergen B, Dodge D, Comanor L (2003). Multicenter evaluation of the VERSANT HCV RNA qualitative assay for detection of hepatitis C virus RNA. J Clin Microbiol.

[70] Gorrin G, Friesenhahn M, Lin P, Sanders M, Pollner R, Eguchi B, Pham J (2003). Performance evaluation of the VERSANT HCV RNA qualitative assay by using transcription-mediated amplification. J Clin Microbiol.

[71] Bortoletto G, Campagnolo D, Mirandola S, Comastri G, Severini L, Pulvirenti FR, Alberti A (2011). Comparable performance of TMA and Real-Time PCR in detecting minimal residual hepatitis C viraemia at the end of antiviral therapy. J Clin Virol.

[72] Jensen DM, Morgan TR, Marcellin P, Pockros PJ, Reddy KR, Hadziyannis SJ, Ferenci P (2006). Early identification of HCV genotype 1 patients responding to 24 weeks peginterferon alpha-2a (40 kd)/ribavirin therapy. Hepatology.

[73] Ferenci P, Laferl H, Scherzer TM, Gschwantler M, Maieron A, Brunner H, Stauber R (2008). Peginterferon alfa-2a and ribavirin for 24 weeks in hepatitis C type 1 and 4 patients with rapid virological response. Gastroenterology.

[74] Lee SS, Sherman M, Ramji A (2010). 36 versus 48 weeks of treatment with peginterferon alfa-2a plus ribavirin for genotype 1/4 patients with undetectable HCV RNA at week 8: final results of a randomized multicenter study. Hepatology.

[75] Fried MW, Hadziyannis SJ, Shiffman ML, Messinger D, Zeuzem S (2011). Rapid virological response is the most important predictor of sustained virological response across genotypes in patients with chronic hepatitis C virus infection. J Hepatol.

[76] Ghany MG, Nelson DR, Strader DB, Thomas DL, Seeff LB (2011). An update on treatment of genotype 1 chronic hepatitis C virus infection: 2011 practice guideline by the American Association for the Study of Liver Diseases. Hepatology.

[77] Jacobson IM, Poordad F, Brown RS, Kwo PY, Reddy KR, Schiff E (2012). Standardization of terminology of virological response in the treatment of chronic hepatitis C: panel recommendations. J Viral Hepat.

[78] Wedemeyer H, Jensen DM, Godofsky E, Mani N, Pawlotsky JM, Miller V (2012). Recommendations for standardized nomenclature and definitions of viral response in trials of hepatitis C virus investigational agents. Hepatology.

[79] Manns MP, von Hahn T (2013). Novel therapies for hepatitis C - one pill fits all?. Nat Rev Drug Discov.

[80] Tran TT (2012). A review of standard and newer treatment strategies in hepatitis C. Am J Manag Care.

[81] Habersetzer F, Leboeuf C, Doffoel M, Baumert TF (2012). Boceprevir and personalized medicine in hepatitis C virus infection. Pharmgenomics Pers Med.

[82] Manns MP, Wedemeyer H, Cornberg M (2006). Treating viral hepatitis C: efficacy, side effects, and complications. Gut.

[83] Muir AJ, Bornstein JD, Killenberg PG (2004). Peginterferon alfa-2b and ribavirin for the treatment of chronic hepatitis C in blacks and non-Hispanic whites. N Engl J Med.

[84] Ge D, Fellay J, Thompson AJ, Simon JS, Shianna KV, Urban TJ, Heinzen EL (2009). Genetic variation in IL28B predicts hepatitis C treatment-induced viral clearance. Nature.

[85] Muir AJ (2013). IL28B in the era of direct-acting antivirals for hepatitis C. J Clin Gastroenterol.

[86] McHutchison JG, Everson GT, Gordon SC, Jacobson IM, Sulkowski M, Kauffman R, McNair L (2009). Telaprevir with peginterferon and ribavirin for chronic HCV genotype 1 infection. N Engl J Med.

[87] Kwo PY, Lawitz EJ, McCone J, Schiff ER, Vierling JM, Pound D, Davis MN (2010). Efficacy of boceprevir, an NS3 protease inhibitor, in combination with peginterferon alfa-2b and ribavirin in treatment-naive patients with genotype 1 hepatitis C infection (SPRINT-1): an open-label, randomised, multicentre phase 2 trial. Lancet.

[88] Hezode C, Forestier N, Dusheiko G, Ferenci P, Pol S, Goeser T, Bronowicki JP (2009). Telaprevir and peginterferon with or without ribavirin for chronic HCV infection. N Engl J Med.

[89] Bacon BR, Gordon SC, Lawitz E, Marcellin P, Vierling JM, Zeuzem S, Poordad F (2011). Boceprevir for previously treated chronic HCV genotype 1 infection. N Engl J Med.

[90] Zeuzem S, Andreone P, Pol S, Lawitz E, Diago M, Roberts S, Focaccia R (2011). Telaprevir for retreatment of HCV infection. N Engl J Med.

[91] Rong L, Dahari H, Ribeiro RM, Perelson AS (2010). Rapid emergence of protease inhibitor resistance in hepatitis C virus. Sci Transl Med.

[92] Sarrazin C, Zeuzem S (2010). Resistance to direct antiviral agents in patients with hepatitis C virus infection. Gastroenterology.

[93] Foster GR, Hezode C, Bronowicki JP, Carosi G, Weiland O, Verlinden L, van Heeswijk R, Vangeneugden T, Picchio G, Beumont-Mauviel M (2009). 50 activity of telaprevir alone or in combination with peginterferon alfa-2a and ribavirin in treatment-naive genotype 2 and 3 hepatitis-C patients: interim results of study C209. J Hepatol.

[94] Kieffer TL, Sarrazin C, Miller JS, Welker MW, Forestier N, Reesink HW, Kwong AD (2007). Telaprevir and pegylated interferon-alpha-2a inhibit wild-type and resistant genotype 1 hepatitis C virus replication in patients. Hepatology.

[95] Susser S, Welsch C, Wang Y, Zettler M, Domingues FS, Karey U, Hughes E (2009). Characterization of resistance to the protease inhibitor boceprevir in hepatitis C virus-infected patients. Hepatology.

[96] Manns MP, Bourliere M, Benhamou Y, Pol S, Bonacini M, Trepo C, Wright D (2011). Potency, safety, and pharmacokinetics of the NS3/4A protease inhibitor BI201335 in patients with chronic HCV genotype-1 infection. J Hepatol.

[97] Gane EJ, Stedman CA, Hyland RH, Ding X, Svarovskaia E, Symonds WT, Hindes RG (2013). Nucleotide polymerase inhibitor sofosbuvir plus ribavirin for hepatitis C. N Engl J Med.

